# Effect of a Six-Week Preparation Period on Acute Physiological Responses to a Simulated Combat in Young National-Level Taekwondo Athletes

**DOI:** 10.1515/hukin-2015-0067

**Published:** 2015-10-14

**Authors:** Pantelis T. Nikolaidis, Hamdi Chtourou, Gema Torres-Luque, Ioannis G. Tasiopoulos, Jan Heller, Johnny Padulo

**Affiliations:** 1 Department of Physical and Cultural Education, Hellenic Army Academy, Athens, Greece.; 2 Exercise Physiology Laboratory, Nikaia, Greece.; 3 Tunisian Research Laboratory “Sports Performance Optimisation” National Center of Medicine and Science in Sports (CNMSS), Tunis, Tunisia.; 4 Faculty of Science, University of Jaen, Spain.; 5 Faculty of Human Movement and Quality of Life Sciences, University of Peloponnese, Sparta, Greece.; 6 Faculty of Physical Education and Sport, Charles University in Prague, Czech Republic.; 7 University e-Campus, Novedrate, Italy.

**Keywords:** anthropometry, martial arts, physical fitness, training load, youth

## Abstract

The aim of this study was to examine changes in physical attributes, physiological characteristics and responses that occurred in a simulated combat during a six-week preparatory period in young taekwondo athletes. Seven athletes (age 12.17 ± 1.11 years) were examined before (pre-intervention) and after (post-intervention) a preparatory period for physical fitness and physiological responses to a 2×90 s simulated bout with a 30 s rest period. The heart rate (HR) was monitored during the simulated combat, and handgrip muscle strength (HMS) along with the countermovement jump (CMJ) were recorded before and after the combat. When compared with pre-intervention values, in post-intervention we observed a decrease in body mass, body fat percentage, and the HR at rest and during recovery after a 3 min step test, and an increase in maximal velocity of the cycle ergometer force-velocity test, the CMJ and mean power during the 30 s continuous jumping test (p<0.05). Furthermore, HR responses to a simulated combat were lower in the post-intervention session (p<0.05). CMJ values increased after the bout in both pre and post-intervention, with higher absolute values in the latter case (p<0.05), whereas there was no difference in HMS. Based on these findings, it can be concluded that the acute physiological responses to a simulated taekwondo combat vary during a season, which might be explained by changes in physical fitness.

## Introduction

Taekwondo is an Olympic combat sport (Bridge et al., 2014) renowned for its kicking techniques (Fong et al., 2013). It is of Korean origin and is practiced in more than 180 countries (Fong and Ng, 2011). Taekwondo is characterized by specific physiological demands, including dynamic phases and powerful kicks (Hammami et al., 2013). Coaches recognize that certain motor and functional skills have a significant impact on sports performance (Čular et al., 2013). International taekwondo athletes possess low levels of body fat, moderate to high levels of cardiorespiratory fitness and high anaerobic power (Bouhlel et al., 2006; Bridge et al., 2014; Chan et al., 2003; Hammami et al., 2013; Hammami et al., 2014; Heller et al., 1998; Lin et al., 2006). Professional taekwondo athletes when compared with amateurs and non-athletes have better neuromotor abilities and faster reactions to sport-specific stimuli (Chung and Ng, 2012). Compared with less successful national-level athletes in this sport, those who were classified as successful have less body fat, are taller, have better aerobic capacity, explosive power and agility (Marković et al., 2005). Taekwondo medalists in the Olympic Games performed more defensive kicks to the trunk and the head than non-medalists (Čular et al., 2011). In the Sydney 2000 Olympic Games, offensive kicks accounted for at least 52% of the techniques for scoring a point, and winners overall tended to be younger and taller, with a slightly lower body mass index than their weight category average (Kazemi et al., 2006).

The physiological profile of taekwondo athletes as reported above corresponds to the metabolic demands of the competition (Campos et al., 2012; Casolino et al., 2012; Tornello et al., 2013). It has been shown that in young adult athletes, the mean ratio of high intensity actions to moments of low intensity (steps and pauses) was 1:7, and the relative contribution of the aerobic, lactic anaerobic and alactic anaerobic energy transfer system was 66, 30 and 4% during a bout (Campos et al., 2012). Moreover, a study on cadet taekwondo athletes showed that a 3×90 s bout with 60 s rest periods was composed of 42% fighting, 45% non-fighting and 13% stoppage time, lasting 2.8 s, 6.5 s and 13.7 s, respectively (Tornello et al., 2013). In 10–12 year old athletes, a variation of offensive and defensive actions according to round was observed (with a greater number of offensive actions in consecutive rounds) (Casolino et al., 2012).

Many studies have examined acute physiological responses to an official or simulated combat or taekwondo training (Bridge et al., 2013; Casolino et al., 2012; Chiodo et al., 2011; Chiodo et al., 2012). For instance, in a comparison between selected and non-selected athletes, the former had better responses to training in terms of rated perceived exertion and lactate concentration (Casolino et al., 2012). In international taekwondo athletes, the championship combat elicited a higher heart rate (HR) than a specific exercise protocol (188 vs. 172 bpm) (Bridge et al., 2013). In addition to the HR and lactate concentration, indices of neuromuscular performance (e.g. the countermovement jump (CMJ), handgrip muscle strength) have also been used as physiological markers during an official or a simulated taekwondo combat (Chiodo et al., 2011; Chiodo et al., 2012). The indices of neuromuscular performance do not follow a unique pattern before and after combat; the CMJ increases, whereas handgrip muscle strength decreases indicating high neuromuscular activation of lower limbs and fatigue of upper limbs due to repeated concussions from the opponent’s kicks and punches directed toward the scoring area of the torso (Chiodo et al., 2011; Chiodo et al., 2012).

Despite the contribution of the aforementioned studies to our understanding of the acute physiological responses to a simulated or official taekwondo combat, to the best of our knowledge no study has ever examined such responses in different periods of an annual season. It would be of special interest to understand the seasonal changes of physiological responses to a simulated bout taking into account that coaches and fitness trainers widely use such combats as exercise interventions. Considering the fact that the level of physical fitness changes during a season (e.g. an increase in jump performance after 12 week training (Ke-tien, 2012), an increase in the height of the squat-jump after 9 week training (Ball et al., 2011) or an increase in muscle strength and a sit-and-reach score after a 12 week program (Kim et al., 2011)), it is reasonable to hypothesize that acute responses to a simulated bout might vary according to changes in physical fitness. Therefore, the aim of the present study was to investigate the effect of the preparatory period on these responses in national-level youth taekwondo athletes.

## Material and Methods

### Participants

**Figure 1:**
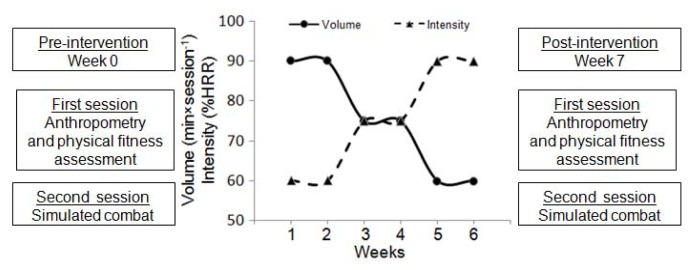
Study design. HRR=heart rate reserve

Seven taekwondo athletes (three females and four males, age 12.17 ± 1.11 years, body mass 46.8 ± 9.2 kg, body height 154.4 ± 7.8 cm and body fat 18.0 ± 4.2%), all members of a sport club and who competed at the national level, volunteered to participate in this study. All participants had at least five years of sports experience and their training during the preparatory period consisted of five sessions per week with each session lasting 60–90 min (Figure 1). They were familiar with the testing procedures as the physical fitness battery used in this study had been routinely administered to the members of this sports club in the past.

To accomplish the aim of the present study, participants were measured in the beginning and at the end of the preparatory period of the 2013–2014 season. Physical fitness components were designated as dependent variables. The preparation period (pre and post-intervention) and the simulated combat (before and after) were designated as the independent variables. The study protocol was performed in accordance with the ethical standards of the Declaration of Helsinki, and was approved by the local Institutional Review Board (Exercise Physiology Laboratory, Nikaia, Greece). Informed consent from the athletes and their parents was obtained.

In both pre and post-intervention, each participant took part in two testing sessions on weekdays, separated by 48-h of recovery. The first session included anthropometric, body composition, flexibility, aerobic capacity, isometric muscle strength and jumping evaluations and a force-velocity test performed in the exercise physiology laboratory. The second session was conducted in the club’s indoor practice center, where the simulated taekwondo combat was performed. On all occasions, measurements were carried out under standard environmental conditions (temperature 22–24°C and humidity 50–54%) between 9 and 11 am. Except for the 30 s Bosco test, the endurance test and the simulated combat, which were performed once, two trials were allowed for the remaining tests and the better score was recorded for further analysis. The intra-class correlation coefficients for the tests ranged from 0.91 to 0.99 (Dal Pupo et al., 2013; MacDonccha et al., 1999).

### Procedures

In the first session, body height, body mass and skinfolds were measured with subjects barefoot and in minimal clothing. An electronic weight scale (HD-351 Tanita, Illinois, USA) was employed for body mass measurement (to the nearest 0.1 kg), a portable stadiometer (SECA, Leicester, UK) for height in the Frankfurt plane (0.1 cm) and a caliper (Harpenden, West Sussex, UK) for skinfold measurement (0.5 mm). The body mass index (BMI) was calculated as the quotient of body mass (kg) to height squared (m^2^), and body fat percentage (BF) was estimated from the sum of 10 skinfolds (cheek, wattle, chest I, triceps, subscapular, abdominal, chest II, suprailiac, thigh and calf; BF = −41.32 + 12.59 × log_e_x, where x is the sum of the 10 skinfolds) (Parizkova, 1978). Chronological age for each participant was calculated using a table of decimals of year (Ross and Marfell-Jones, 1991).

Thereafter, the participants were measured for the resting HR in a supine position for 5 min (Aubert et al., 2003). The HR was recorded continuously during all testing procedures in the laboratory and in the field by Team2 Pro (Polar Electro Oy, Kempele, Finland). Physical working capacity at HR 170 bpm (PWC_170_) was evaluated on a cycle ergometer (828 Ergomedic, Monark, Sweden) according to the Eurofit guidelines (Bland et al., 2012). Seat height was adjusted to each participant’s satisfaction, and toe clips with straps were used to prevent the feet from slipping off the pedals. The participants were given instructions before the test to pedal with a steady cadence of 60 rpm, which was provided by both visual (the ergometer’s screen showing a pedaling cadence) and audio means (a metronome set at 60 bpm). The test consisted of three stages, each lasting 3 min, against incremental braking force, in order to elicit a HR between 120 and 170 bpm. Based on the linear relationship between the HR and power output, PWC_170_ was calculated as the power corresponding to the HR of 170 bpm and expressed as W and W·kg^−1^. In addition to PWC_170_, the participants also performed a 3 min step test (Nikolaidis, 2011), in which they ascended and descended using a 24 ascent·min^−1^ cadence against a step height of 30 cm. The HR was recorded at the end of this test as well as at the end of the first minute of recovery.

#### The sit-and-reach test (SAR)

The sit-and-reach test (SAR) (Ayala et al., 2012) was employed for the assessment of low back and hamstring flexibility. An advantage of 15 cm was set at the position of just reaching the toes. Isometric strength testing included two measures: right handgrip and left handgrip (Heyward, 2010). In the handgrip test, after the dynamometer was fitted to the hand being tested so that the bar was resting on the phalanx of the index and ring finger, the participants were instructed to squeeze the handle of a handgrip dynamometer (Takei, Tokyo, Japan) as hard as possible, while standing with their elbow bent at approximately 90°.

#### The CMJ and 30 s Bosco test

The participants performed the CMJ with the arm swing (Aragon-Vargas, 2000). They started in a standing position with both feet together and were asked to jump as high as possible with a rapid countermovement. The depth of the countermovement was self-selected, and participants were asked to land as close as possible to their point of take-off (Padulo et al., 2013). Flight time was used to calculate the change in the height of the body’s centre of gravity (Bosco et al., 1983). The height of the jump was estimated using the Opto-jump (Microgate Engineering, Bolzano, Italy). The Bosco test was conducted on the same equipment as the CMJ. The athletes were instructed to jump continuously for 30 s as high as possible, while trying to stay on the ground as little as possible (Sands et al., 2004). Mean power was recorded in W·kg^−1^.

#### The force-velocity test

The force-velocity test (Driss and Vandewalle, 2013) was used to assess maximal power (P_max_) expressed in W and W·kg^−1^, theoretical maximal velocity (v_0_) and force (F_0_). This test employed various braking forces that elicited different pedaling velocities in order to derive Pmax. The participants performed four sprints on a leg cycle ergometer (Ergomedics 874E, Monark, Sweden) with an upper handlebar (Padulo et al., 2012), each one lasting 7 s, against an incremental braking load (19.6, 29.4, 39.2 and 49.0 N), interspersed by 5 min recovery periods.

In the second session, the participants performed a 2×90 s simulated taekwondo combat, with 30 s passive rest periods. During the combat, the athletes were encouraged vigorously to reach maximal performance. Within a minute before and after the combat, they were tested for the CMJ and handgrip muscle strength in both hands. Two trials were allowed for the CMJ and handgrip strength test (Laffaye et al., 2014) (REF), and the better result was recorded. The HR of athletes was monitored continuously during the two rounds and the break, and the corresponding mean values were recorded for further analysis. In addition to the absolute values of the HR, we also calculated exercise intensity as percentage of the HR reserve (%HRR), based on the Karvonen method (Karvonen et al., 1957). This method takes into account the HR at rest, which had already been measured in the laboratory, and the maximal HR, which was calculated based on the Tanaka formula (208-0.7×age) (Tanaka et al., 2001). Accordingly, exercise intensity was estimated as %HRR=100×(HRexercise-HRrest)/(HRmax-HRrest).

### Statistical analysis

Statistical analyses were performed using IBM SPSS v.20.0 (SPSS, Chicago, USA). Data were expressed as mean and standard deviations (*SD*). Only the better score of each test was included in the data analysis, and parametric analysis techniques were used. A two-way repeated measures analysis of variance (ANOVA) with a subsequent Bonferroni post-hoc test (if differences between groups were revealed) was used to examine differences between the simulated combats on two occasions (pre- and post-intervention). To interpret effect sizes (ES) for statistical differences in the ANOVA, we used eta square classified as small (0.01<η^2^≤0.06), medium (0.06<η^2^≤0.14) and large (η^2^>0.14) (Cohen, 1988). A student independent t-test was employed to test differences between pre and post-intervention. The level of significance was set at *α*=0.05.

## Results

The physical fitness components before and after the six-week preparation period are presented in Table 1. Compared with pre-intervention values, we observed a decrease in body mass, body fat percentage, the body mass index, and the HR at rest and during recovery after a 3 min step test, as well as an increase in the sit-and-reach score, maximal velocity of the cycle ergometer force-velocity test, the CMJ and mean power during the 30 s continuous jumping test (p<0.05). With regard to the simulated taekwondo combat, there was a significant main effect of the preparation period on the HR (F_1,6_=9.0, p=0.024, η^2^=0.60) and the CMJ (F_1,6_=7.2, p=0.036, η^2^=0.55), but not on the HRR (F_1,6_=4.3, p=0.085, η^2^=0.42) or right (F_1,6_=2.7, p=0.153, η^2^=0.31) and left handgrip muscle strength (F_1,6_=4.6, p=0.076, η^2^=0.43). Furthermore, we observed a significant main effect of the simulated combat on the HR (F_1,6_=661.3, p<0.001, η^2^=0.99), the HRR F_2,12_=27.4, p<0.001, η^2^=0.82) and the CMJ (F_1,6_=8.6, p=0.026, η^2^=0.59), but not on right (F_1,6_=2.2, p=0.188, η^2^=0.27) and left handgrip muscle strength (F_1,6_=0.7, p=0.450, η^2^=0.10). Moreover, there was a significant interaction between the preparatory period and a simulated combat on the HR (F_1,6_=7.1, p=0.037, η^2^=0.54) and the CMJ (F_1,6_=8.3, p=0.028, η^2^=0.58), but not on the HRR (F_2.12_=1.6, p=0.244, η^2^=0.21) or right (F_1,6_=1.9, p=0.218, η^2^=0.24) and left handgrip muscle strength (F_1,6_=0.4, p=0.532, η^2^=0.07).

**Figure 2:**
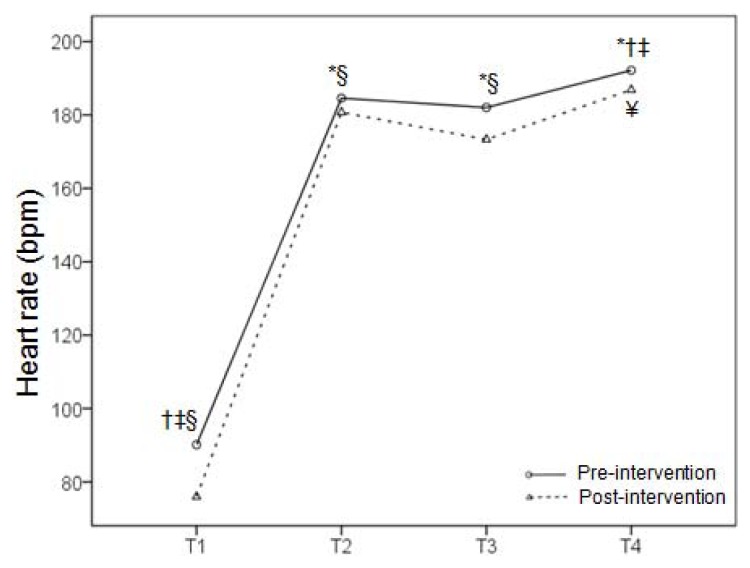
Mean heart rate at rest (T1), during the first half of the simulated combat (T2), during the rest period (T3) and during the second half (T4) in the beginning (pre-intervention) and at the end (post-intervention) of the preparatory period. The symbols *, †, ‡ and § denote a significant difference among T1, T2, T3 and T4, respectively, whereas ¥ denotes a difference between pre-intervention and post-intervention.

**Figure 3:**
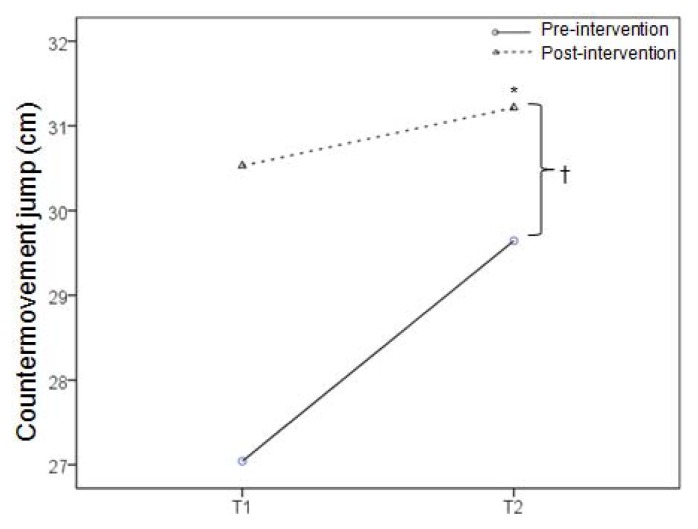
Countermovement jump before (T1) and after (T2) a simulated combat in the beginning (pre-intervention) and at the end (post-intervention) of the preparatory period. The symbols * and † denote a significant difference between T1 and T2, and between pre-intervention and post-intervention, respectively.

**Figure 4:**
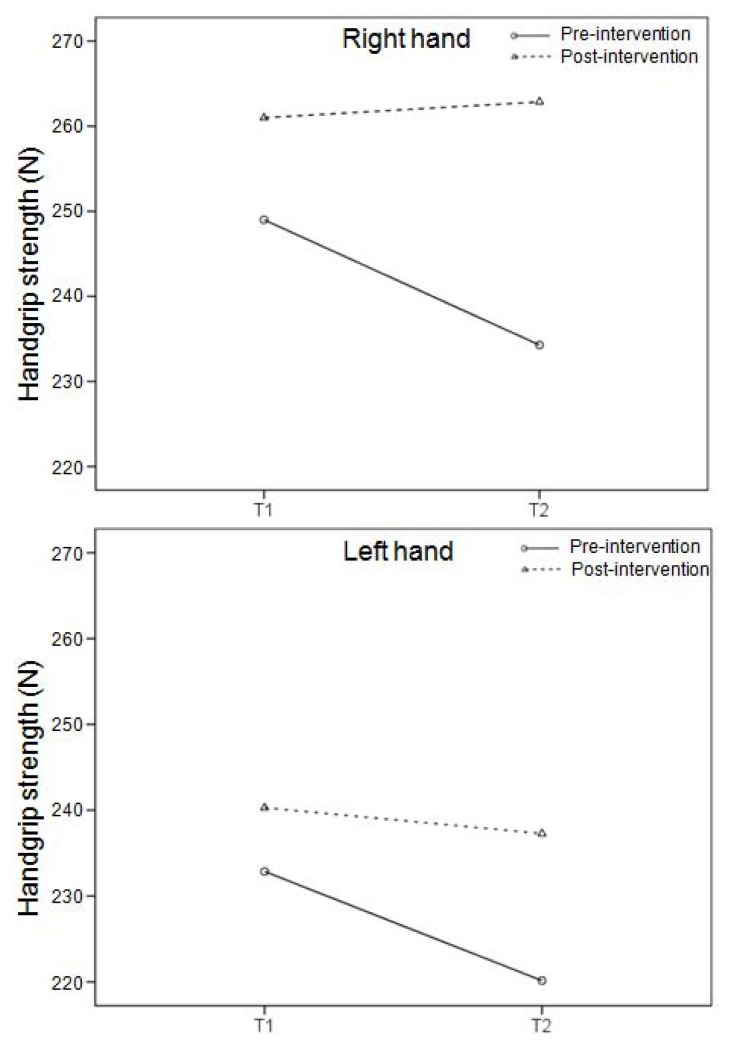
Handgrip muscle strength of the right (A) and left (B) hand before (T1) and after (T2) a simulated combat in the beginning (pre-intervention) and at the end (post-intervention) of the preparatory period.

During the taekwondo combat, the highest mean HR was registered in the second round, whereas there was no difference in the HR between the first round and the break (Figure 2). This trend was observed in both the pre and post-intervention; however, all HR values were lower in the latter case. A mean HR during the simulated combat in pre-intervention was 184.6 ± 10.0, 182.0 ± 7.9 and 192.1 ± 9.8 bpm in the first round, during the break and in the second round, respectively, whereas corresponding values in post-intervention were 180.7 ± 8.8, 173.3 ± 11.2 and 186.9 ± 9.5 bpm. These absolute HR values corresponded to exercise intensity of 86.6 ± 8.4, 84.1 ± 6.0 and 93.7 ± 8.6% of the HRR in pre-intervention, and 85.0 ± 6.8, 79.1 ± 7.9 and 89.9 ± 7.4% of the HRR in post-intervention. The CMJ performance was improved after the combat in both pre (2.6 cm) and post-intervention (0.7 cm), with higher absolute values in the latter case (p<0.05) (Figure 3), whereas there was no difference in handgrip muscle strength (Figure 4).

## Discussion

This is one of the first studies to examine physiological responses to a simulated taekwondo bout during the preparatory period. The main finding was the significant interaction between the preparatory period and the simulated combat on the HR and the CMJ. First, physical fitness was tested before and after this period, and the results showed an increase in most of the examined variables. Considering that taekwondo athletes compete in weight categories, significant changes in body mass and BF highlighted importance of this period for the achievement of optimal body mass for official competitions.

To evaluate aerobic capacity, we used two measures relying on the HR response to the submaximal load – either on a cycle ergometer (PWC170) or a step test, and we analyzed two indices for each test. Among the four indices of aerobic capacity, we found significant improvement only in the HR at the end of the first minute of recovery after the step test. Although non-significant, the other indices also indicated an improvement in aerobic capacity. The increase in aerobic capacity results from the augmented aerobic training, which underpins sport preparation. Despite the nature of taekwondo being a sport that includes high-intensity and short duration actions, aerobic training in the preparatory period stimulates an increased activity of the parasympathetic nervous system, which is evident from the significant decrease in the resting HR.

The force-velocity test was administered on a cycle ergometer to measure anaerobic power. Compared with other anaerobic power tests (e.g. the Wingate anaerobic test), this test provides additional information about the components of power (i.e. force and velocity), and its duration corresponds to the duration of high-intensity activities during a combat. We did not find any difference between pre and post-intervention when power was expressed in W or W·kg−1. However, maximal velocity increased during the preparatory period, indicating a positive effect of taekwondo training on this physical fitness component. This observation is in agreement with previous research that compared athletes with a different number of training hours per week, where a positive correlation between duration of taekwondo practice and isokinetic muscle strength of both the quadriceps and hamstrings at fast speeds (240°.s−1), but not at slow speeds (60°·s−1), was observed (Fong and Tsang, 2012).

In turn, an increased activity of the parasympathetic nervous system lowers HR responses during exercise, which was observed in the simulated combat. In addition to the HR, we also examined the HRR, which in contrast to the absolute values of the HR did not reveal any significant main effect of the preparatory period nor an interaction between the preparatory period and the simulated bout. This discrepancy may be attributed to the decrease in the HRrest in the post-intervention session. The changes during the preparatory period should be attributed to the training loads, which characterized this period. A previous study on exercise intensity during the preparatory period revealed that most of the time was spent at intensities between 60 and 90% of the HRmax, and only 10% was spent above 90% of the HRmax (Haddad et al., 2014).

The simulated taekwondo combat in this study elicited a very high intensity load (85–94% of HRR). This value was higher than that reported in studies on the assessment of intensity of various sport-specific exercises. For instance, repeated kicks of four bouts of 10 s with a 20 s break elicited ~71% of the HRR (Haddad et al., 2011). Moreover, in a comparison of two beginners’ forms (one with only arm techniques and one with both arm and leg techniques) and two sets of technique combinations (one set that consisted of kicks only and the other of kicks and punches), the HR for both forms was 80% of the HRmax, while that of the combinations was ~ 90% of the HRmax (Pieter et al., 1990). In a study of eight fundamental taekwondo training activities (technical combinations, step sparring, pad work, forms, basic techniques and forms, sparring drills, and free sparring), the HR varied from 65 to 81% of the HRmax (Bridge et al., 2007). Thus, a simulated combat might be considered an alternative training tool that can provide higher intensities than other sport-specific exercises. Another aspect of the simulated combat was that the intensity was not distributed evenly between the two rounds, and it achieved the highest value in the second one. This is not the first time such a variation has been reported in the exercise intensity during a combat. For instance, Bridge and colleagues (Bridge et al., 2009) observed an increase in the HR from round one to round three, and Heller and colleagues (Heller et al., 1998) found that the HR was 184 and 186 bpm in the first and second round, respectively, in a 2×120 s official combat. Although, we would expect a lower exercise intensity due to reduced stress response, the simulated combat in the present study seemed to elicit an intensity similar to that of official combats (Marković et al., 2008; Matsushigue et al., 2009).

The findings of this study provide a novel insight into the exploration of the impact of a simulated taekwondo combat on various physiological variables, as it was the first research analyzing this impact on two separate occasions. However, a limitation of this study was that data were collected only in the beginning and at the end of the preparatory period, whereas future research should consider examining this impact on more occasions during an annual training cycle, including the competitive period. It would be interesting for a future study to examine the relationship between performance and physiological changes during an official combat as the findings of a recent study on another martial art sport (kickboxing) revealed that a decrease in handgrip muscle strength was related to the outcome of the combat (Tasiopoulos and Nikolaidis, 2013). In addition, the actual age (~12 years) of our participants should be considered in the interpretation of the findings. Although the biological age was not considered in the analysis of the results, a trivial change in body height (0.2 cm) during the six-week preparation period indicated a lack of maturation effect on the variation in physiological responses. Caution is needed when applying our findings to older athletes, as it is assumed that athletes of a higher sports level (e.g. national-level adults) possess greater aerobic and anaerobic capacities and have a smaller variation in their physical fitness during a season, thus, consequently a smaller variation in physiological responses to a simulated bout should be expected.

Based on the findings of the present study, it can be concluded that the acute physiological responses of young athletes to a simulated taekwondo combat vary during a season. This variation might be explained by corresponding changes in physical fitness. Therefore, coaches and fitness trainers should be aware that the same exercise intervention might elicit different physiological responses depending on a particular period within the annual training cycle.

## Figures and Tables

**Table 1:** Physical fitness in youth taekwondo athletes before (pre-intervention) and after (post-intervention) a six week preparatory period.

	Pre-intervention	Post-intervention	Difference (mean (95% CI))
	
Body mass (kg)	46.8±9.2	45.2±9.0*	−1.5 (−2.6; −0.4)
Body height (cm)	154.4±7.8	154.5±8.0	0.2 (−0.4; 0.7)
BMI (kg·m^−2^)	19.5±2.3	18.8±2.3*	−0.7 (1.1; −0.2)
BF (%)	18.0±4.2	15.2±4.4‡	−2.8 (−4.0; −1.6)
SAR (cm)	22.4±7.1	25.8±5.9*	3.4 (0.8; 6.0)
HRrest (bpm)	90±14	76±10*	−14 (−25; −4)
PWC_170_ (W)	97±19	98±16	1 (−8; 10)
PWC_170_ (W·kg^−1^)	2.09±0.21	2.21±0.29	0.12 (−0.09; 0.33)
Step_1_ (bpm)	156±5	148±9	−8 (−19; 3)
Step_2_ (bpm)	117±15	101±16*	−16 (−31; 0)
P_max_ (W)	536±134	550±128	14 (−86; 114)
P_max_ (W·kg^−1^)	11.5±2.0	12.2±1.8	0.7 (−1.5; 2.9)
V_0_ (rpm)	156±26	177±20†	21 (9; 33)
F_0_ (N)	139±33	124±24	−15 (−45; 15)
RH (N)	239±47	252±43	13 (−20; 46)
LH (N)	231±43	232±45	1 (−14; 16)
Sum (N)	470±80	484±86	14 (−24; 52)
Sum (N·kg^−1^)	10.2±1.4	10.9±2.0	0.7 (−0.2; 1.6)
CMJ (cm)	27.2±4.8	29.9±5.2†	2.7 (1.5; 4.0)
Bosco (W·kg^−1^)	25.9±5.2	34.9±8.1‡	9.0 (5.6; 12.4)

BMI=body mass index, BF=body fat percentage, SAR=sit-and-reach test, HRrest=heart rate at rest, PWC_170_=physical working capacity in heart rate 170 bpm, Step_1_=heart rate in the end of step test, Step_2_=heart rate in the end of the first minute of recovery after step test, P_max_=maximal power, V_0_=maximal velocity, F_0_=maximal force, RH=right handgrip muscle strength, LH=left handgrip muscle strength, Sum=sum of right and left handgrip muscle strength, CMJ=countermovement jump, Bosco=mean power during a 30 s continuous jumping test.

* p<0.05,

† p<0.01,

‡ p<0.001.
